# Abdominal aortic calcification score can predict future coronary artery disease in hemodialysis patients: a 5-year prospective cohort study

**DOI:** 10.1186/s12882-018-1124-x

**Published:** 2018-11-08

**Authors:** Hung-Chih Chen, Wei-Ting Wang, Chieh-Ning Hsi, Che-Yi Chou, Hsuan-Jen Lin, Chiu-Ching Huang, Chiz-Tzung Chang

**Affiliations:** 1 Division of Nephrology, Asia University Hospital, Taichung, Taiwan; 2 College of Medicine, China Medical University, Taichung, Taiwan; 3 Division of Nephrology, China Medical University Hospital, Taichung, 40447 Taiwan

**Keywords:** Abdominal aortic calcification, Coronary artery disease, Hemodialysis

## Abstract

**Background:**

Abdominal aortic calcification (AAC) has been known to be associated with cardiovascular mortality in hemodialysis. However, the association between AAC and future coronary artery disease (CAD) occurrence is not clear. We aimed to clarify the association of AAC severity and the occurrence of future CAD events in hemodialysis patients.

**Methods:**

Hemodialysis (HD) patients were recruited in this prospective cohort study. AAC severity was quantified by AAC score, which was measured by lateral lumbar radiography. We used receiver operation curve (ROC) analysis to find the cutoff AAC value for CAD prediction. CAD-free survival was analyzed by Kaplan-Meier study.

**Results:**

There were 303 patients recruited for study with a median (interquartile range) follow-up of 95 (65–146) months. The AAC score in patients with occurrence of new CAD [9 (3–15.25), *n* = 114] was higher than in patients without new CAD occurrence [5 (1–9) *n* = 189], *p* < 0.001. Multivariate hazard ratio of AAC score for CAD was 1.039 (*p* = 0.016). ROC study showed that an AAC score of 5.5 had a sensitivity of 0.658 and a specificity of 0.587 in the prediction of new CAD occurrence. Patients with AAC score above 5.5 had significantly higher cumulative incidence of CAD than patients with AAC score below 5.5. Age, diabetes, prior history of CAD, and longer dialysis vintage were major factors associated with higher AAC score.

**Conclusions:**

AAC score can predict the occurrence of future CAD events in HD patients. The best cut-off value of AAC score is 5.5. AAC score greater than 5.5 is a reliable abdominal aortic calcification marker, and can predict future CAD in ESRD patients. Major contributive factors for higher AAC score were age, presence of diabetes, prior history of CAD, and longer dialysis vintage.

## Background

Vascular calcification is commonly seen in chronic kidney disease (CKD) patients [1, 2]. The prevalence of vascular calcification in end stage renal disease patients can be as high as 81% in a cross-sectional study [3]. The Kidney Disease Improving Global Outcome (KDIGO) guideline has recommended routine lateral plain radiograph in CKD stage 3-5D patients since 2009 [4]. A plain lateral lumbar graphic film can provide possible information about CKD-MBD (Mineral Bone Disease) status, vascular calcification, and cardiovascular risk for patients. Abdominal aortic calcification had been used as an indicator of vascular calcification of hemodialysis patients in previous studies [5]. Presence of abdominal aortic calcification (AAC) was associated with higher cardiovascular mortality and can be a novel marker for atherosclerosis risk in hemodialysis patients [5, 6]. However, using severity of AAC to predict coronary artery disease (CAD) in chronic HD patients has not yet been investigated. The severity of AAC can be measured by a semi-quantitative method or AAC score as proposed by Kauppila LI et al. in 1977 [7]. We conducted a 5-year prospective cohort study in chronic HD patients to analyze the relationship between AAC score and future CAD events.

## Methods

End-Stage Renal Disease (ESRD) patients on chronic hemodialysis (dialysis vintage longer than 3 months) in China Medicine University Hospital were included in this prospective cohort study. We recruited hemodialysis patients on maintenance hemodialysis from January 2013 to April 2018. Patients’ baseline abdominal aortic calcification scores were measured by lateral lumbar x-ray in the day of recruitment. Two physicians blind to patients’ clinical information performed the AAC score calculation via a 24-point scoring system developed by Kauppila LI et al. [7]. Calcification scores of the anterior and posterior wall of abdominal aorta over 1st lumbar spine to 4th lumbar spine (L1 to L4) were recorded. Calcification score of each section ranges from 0 to 3. Score 0 represents without any calcification; score 1 represents calcification length less than 1/3 of vertebra; score 2 represents the calcification length spanned from 1/3 to 2/3 of vertebra; score 3 represents calcification length greater than 2/3 of vertebra. AAC score is the sum of calcification score from L1 to L4 with a maximum of 24. AAC score 0 represents no abdominal aortic calcification in lateral lumbar x-ray and AAC score 24 represents a most severe degree of abdominal aortic calcification in lateral lumbar x-ray.

All patients underwent hemodialysis treatment in a 3.5–4 h session bi-weekly or thrice-weekly, using high-flux artificial kidneys with low molecular weight heparin as anticoagulant. The quality of water for hemodialysis was monitored regularly following the standards of Association for the Advancement of Medical Instrument (AAMI) guidelines for dialysis water. Patients’ clinical laboratory data, including serum albumin, creatinine, uric acid, total cholesterol, triglyceride, fasting blood sugar, calcium, phosphate, sodium, potassium, alanine aminotransferase (ALT), alkaline phosphatase (ALKP), white cell count, hemoglobin, intact parathyroid hormone (iPTH), ferritin, body mass index (BMI), and spKt/V were obtained at recruitment day. Dialysis adequacy, spKt/V, was measured by the second-generation single-pool Daugirdas formula [8]. Diabetes mellitus (DM) was defined as hypoglycemic agents using or a fasting blood sugar level of 126 mg/dl or more [9]. Hypertension was defined as antihypertensive agents use, systolic blood pressure greater than 140 mmHg, or diastolic blood pressure greater than 90 mmHg [10]. CAD was defined by the presence of angina, electrocardiographic changes, and elevation of cardiac enzymes [11]. Coronary angiography was performed for all the CAD patients. Follow up time defined as from patient recruitment to CAD occurrence or patient mortality or the ending of the study follow up set at April 2018. The study was approved by the China Medical University & Hospital Research Ethics Committee, Taiwan (Reference number: CMUH-104-REC1–110). Blood sampling and lumbar spine x-ray from all study subjects was performed after obtaining informed consent.

## Statistical analysis

Continuous data passing normality test were reported as mean ± standard deviation, continuous data failed to pass normality test were reported as median with interquartile range. Parametric variables were analyzed by Student’s *t* test, and non-parametric variables were analyzed by Kolmogorov-Smirnov test. Chi-square test was applied for categorical variables. A *p*-value < 0.05 was considered statistically significant. The association between AAC score and factors related to CAD were analyzed using univariate Cox regression model. Associated confounders with a *p* < 0.05 were further analyzed by multivariable Cox regression. ROC (receiver operative curve) curve analysis found the best cutoff value of AAC for CAD prediction. CAD-free survival rate in groups with high or low AAC score were plotted into Kaplan-Meier survival curves. Factors associated with higher AAC score group and non-traditional vascular calcification markers (serum calcium, phosphate, iPTH, clearance of dialysis) were analyzed by multivariate linear regression model. All analyses were performed using SPSS for windows version 18 (IBM Corporation, Chicago, IL, USA).

## Results

We recruited 303 ESRD patients on chronic HD for this study. The demographic data and baseline biochemistries are shown in Table 1. The median age of these patients was 63 (56–71) years old, and 51.5% (*n* = 156) among them were females. The proportion of patients with diabetes was 47% (*n* = 142), with hypertension was 81.5% (*n* = 247), and with prior history of coronary artery disease was 27% (*n* = 82). The medium value of HD vintage was 95 (65–146) months and the median follow-up duration was 68.5 (39.7–68.5) months. The medium value of AAC score in all these patients was 6.0 (2.0–12.0). Acute CAD events occurred in 37.6% (*n* = 114) of the patients during follow-up period. All cause-mortality rate was 22.1% (*n* = 67) while 42% (9.2% (*n* = 28)) of death were cardiovascular-related mortality (CAD, sudden cardiac arrest and peripheral arterial occlusive disease with infection).

**Table 1:** Demographic parameters of hemodialysis patients without and with occurrence of CAD event during follow-up period

	Without CAD	With CAD	
	*n* = 189	*n* = 114	*p*
Age(years old)	62.1 (54.1–71.0)	64.0 (59.0–71.0)	0.114
Male (n, %)	83 (44%)	64 (56%)	**0.039**
DM (n, %)	73 (39%)	69 (61%)	**< 0.001**
HTN (n, %)	152 (80%)	95 (83%)	0.527
CAD history (n, %)	23 (12%)	59 (52%)	**< 0.001**
Vintage (months)	95.0 (64.0–147.0)	95.0 (65.8–141.8)	0.853
BMI (kg/m^2^)	22.1 (19.9–24.1)	23.5 (21.4–25.4)	**0.001**
Hemogloblin (gm/dl)	10.9 (10.1–11.5)	10.7 (10.1–11.5)	0.770
White blood cell (10^3^/uL)	6.3 (4.7–7.9)	6.5 (5.7–7.8)	0.053
Albumin (gm/dl)	4.1 (3.9–4.3)	4.1 (3.9–4.3)	0.786
Creatinine(mg/dl)	10.6 (9.3–12.1)	10.6 (9.2–12.4)	0.841
ALT (U/L)	15 (11–22)	14 (11–19)	0.169
ALKP (U/L)	72 (55–98)	71 (56–89)	0.294
Uric acid (mg/dl)	6.6 (5.8–7.4)	6.6 (5.8–7.3)	0.694
FBS (mg/dl)	89 (79–111)	96 (83–130)	**0.018**
Cholesterol (mg/dl)^a^	165.5 ± 37.9	152.5 ± 35.9	**0.003**
Triglyceride (mg/dl)	106 (71–161)	112 (84–175)	0.329
Calcium (mg/dl)^a^	9.47 ± 0.84	9.47 ± 0.80	0.973
Phosphate (mg/dl)	5.3 (4.5–6.0)	5.7 (4.6–6.8)	**0.048**
iPTH (pg/mL)	184 (61–337)	176 (69–356)	0.907
Ferritin (ng/mL)	486 (318–642)	482 (280–613)	0.341
Sodium(mEq/L)	137 (134–139)	137 (135–139)	0.442
Potassium(mEq/L)	5.0 (4.5–5.5)	5.0 (4.4–5.6)	0.929
spKt/V	1.39 (1.24–1.52)	1.27 (1.14–1.44)	**< 0.001**
AAC score	5 (1–9)	9 (3–15.25)	**< 0.001**

Abbreviations: *AAC score* abdominal aortic calcification score, *DM* diabetes mellitus, *HTN* hypertension, *CAD* coronary artery disease, *BMI* body mass index, *ALT* alanine aminotransferase, *ALKP* alkaline phosphatase, *FBS* fasting blood sugar, *Calcium* serum total calcium (corrected by albumin), *iPTH* intact-parathyroid hormone, *spKt/V*: Single-pool Kt/V

^a^Parametric variables (include serum cholesterol, calcium); except this two variables, all of other continuous variables else are nonparametric

**Fig. 1:**
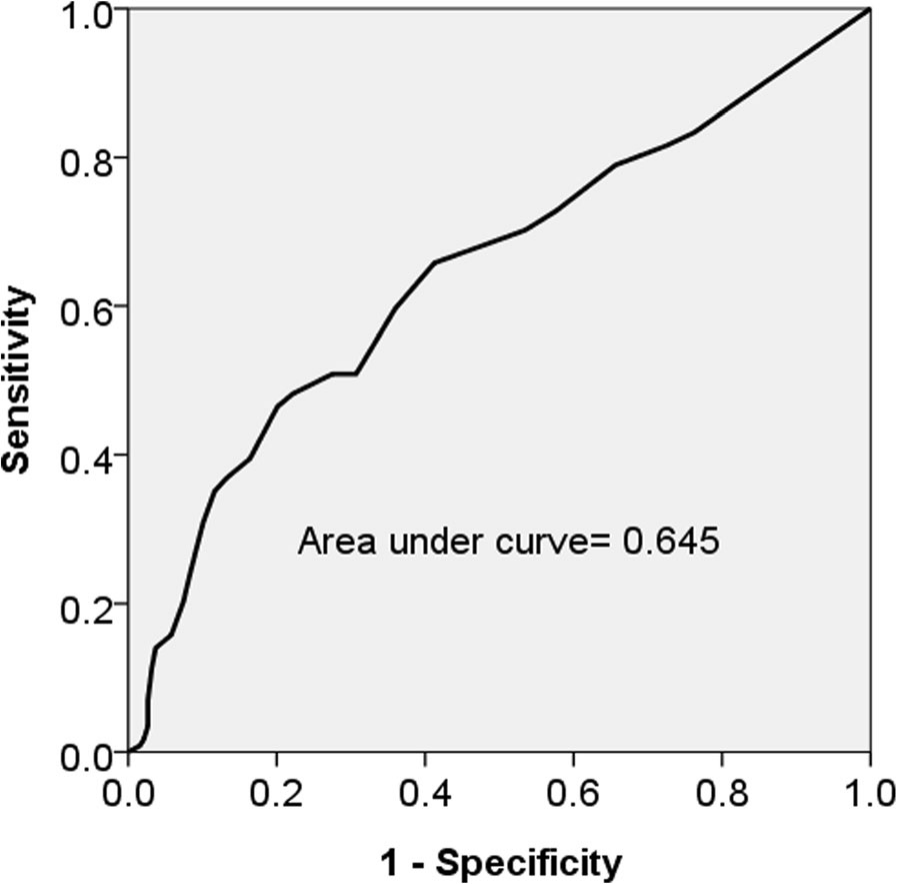
Receiver operating charactiristic (ROC) analysis of abdominal aortic calcification (AAC) score and the risk of future coronary artery disease (CAD). ROC study showed an area under curve of 0.645 (*p* < 0.001). An AAC score grear than 5.5 was associated with future CAD occurence with a sensitivity of 0.658 and a specificity of 0.587

**Fig. 2:**
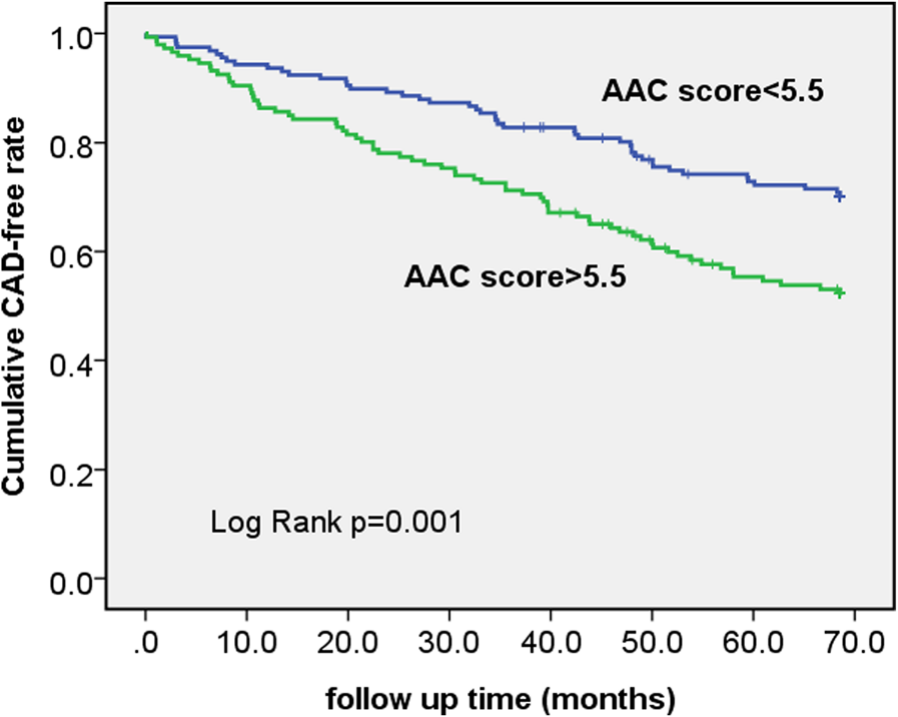
CAD-free survival of patients with AAC score below and above 5.5. Kaplan-Meier study showed hemodialysis dialysis ptients with AAC score lower than 5.5 had a higher cumulative CAD-free survival than that of patients with AAC score higher than 5.5 (*p* = 0.001, log-rank test)

The AAC score in the group with new CAD occurrence (9 (3–15.25), n = 114) was higher than in the group without CAD occurrence (5 (1–9), *n* = 189) (*p* < 0.001). The multi-variate hazard ratio of AAC score for CAD group was 1.039 (*p* = 0.016) (Table 2). ROC analysis found the best AAC cutoff value for CAD prediction—an AAC score of 5.5 had a sensitivity of 0.658 and a specificity of 0.587 in the prediction of future CAD occurrence. (Figure 1) Area under curve of AAC score and CAD occurrence was 0.645 (p < 0.001). Patients with AAC score above 5.5 had higher cumulative incidence of CAD than patients with AAC score below 5.5 (log-rank test, *p* = 0.001) (Fig. 2).

**Table 2:** Hazard ratios of different variable on occurrence of CAD

	Model 1		Model 2	
Variable	HR (95% CI)	p	HR (95% CI)	p
AAC (per 1 score increase)	1.071 (1.043–1.099)	**< 0.001**	1.039 (1.007–1.071)	**0.016**
CAD history (Ref: no)	5.314 (3.659–7.718)	**< 0.001**	3.816 (2.531–5.752)	**< 0.001**
Age (per 1 year old increase)	1.016 (1.000–1.032)	**0.045**	1.013 (0.994–1.032)	0.185
Gender (Ref: female)	1.510 (1.043–2.186)	**0.029**	1.162 (0.766–1.763)	0.481
DM (Ref: no)	2.089 (1.434–3.042)	**< 0.001**	1.359 (0.878–2.102)	0.169
HTN (Ref: no)	1.156 (0.706–1.892)	0.564	0.921 (0.550–1.543)	0.754
Vintage (per 1 month increase)	1.000 (0.997–1.003)	0.985	1.001 (0.998–1.005)	0.415
BMI (per 1 kg/m^2^ increase)	1.071 (1.020–1.124)	**0.006**	1.077 (1.015–1.142)	**0.013**
FBS (per 1 mg/dl increase)	1.003 (1.001–1.006)	**0.020**	1.000 (0.996–1.003)	0.913
Cholesterol (per 1 mg/dl increase)	0.992 (0.987–0.997)	**0.002**	0.996 (0.990–1.001)	0.088
Phosphate (per 1 mg/dl increase)	1.148 (1.012–1.303)	**0.032**	1.090 (0.947–1.254)	0.231
spKt/V (per 1 unit increase)	0.244 (0.108–0.552)	**0.001**	0.502 (0.186–1.349)	0.172

Model 1: crude hazard ratio; Model 2: adjusted for all variables

Abbreviations: *AAC score* abdominal aortic calcification score, *DM* diabetes mellitus, *HTN* hypertension, *CAD* coronary artery disease, *BMI* body mass index, *FBS* fasting blood sugar, *spKt/V* Single-pool Kt/V

Linear regression analysis was further used to identify factors significantly associated with higher AAC score. In our study group, possible cofounders for higher AAC score such as advanced age, DM, hypertension, prior history of CAD, longer dialysis vintage, high serum fasting blood sugar, triglyceride and lower serum creatinine level were analyzed. (Table 3) Other non-traditional vascular calcification contributive factors such as serum calcium, phosphate, iPTH and dialysis clearance marker (spKt/V) were also considered and put into multivariate linear regression analysis. Advanced age (*p* < 0.001), presence of DM (*p* = 0.006), with prior history of CAD (*p* = 0.002) and longer dialysis vintage (*p* = 0.001) were major factors associated with higher AAC group (AAC score > 5.5) (Table 4).

**Table 3:** Demographic parameters of hemodialysis patients with AAC score below and above 5.5

Group	AAC < 5.5	AAC > 5.5	
	*n* = 157	*n* = 146	*p*
Age(years old)	61.0 (53.0–68.0)	65.0 (60.0–73.3)	**< 0.001**
Male (n, %)	81 (52%)	66 (45%)	0.266
DM (n, %)	57 (36%)	85 (58%)	**< 0.001**
HTN (n, %)	127 (81%)	120 (82%)	0.771
CAD history (n, %)	26 (17%)	56 (38%)	**< 0.001**
Vintage (months)	88.0 (62.5–145.0)	102.5 (75.8–148.0)	**0.029**
BMI (kg/m^2^)	22.4 (20.3–24.5)	22.8 (20.7–24.6)	0.965
Hemogloblin (gm/dl)	10.8 (10.1–11.4)	10.9 (9.9–11.6)	0.915
White blood cell (10^3^/uL)	6.3 (4.9–7.8)	6.7 (5.5–8.0)	0.177
Albumin (gm/dl)	4.1 (3.9–4.3)	4.1 (3.9–4.3)	0.755
Creatinine(mg/dl)	11.0 (9.8–12.6)	10.2 (9.1–11.6)	**0.002**
ALT (U/L)	15 (11.5–21.5)	14 (11–20)	0.521
ALKP (U/L)	71 (51–93)	73 (58–92)	0.349
Uric acid (mg/dl)	6.6 (5.9–7.4)	6.6 (5.8–7.3)	0.679
FBS (mg/dl)	90 (78–109)	93 (82–125)	**0.029**
Cholesterol (mg/dl)^a^	162 ± 35	159 ± 40	0.482
Triglyceride (mg/dl)	103 (63–154)	112 (83–174)	**0.028**
Calcium (mg/dl)^a^	9.46 ± 0.86	9.48 ± 0.79	0.810
Phosphate (mg/dl)	5.4 (4.5–6.2)	5.5 (4.6–6.5)	0.320
iPTH (pg/mL)	184 (60–328)	177 (68–362)	0.609
Ferritin (ng/mL)	478 (271–631)	496 (356–645)	0.346
Sodium(mEq/L)	137 (135–139)	137 (134–138)	0.404
Potassium(mEq/L)	4.9 (4.5–5.5)	5.0 (4.5–5.6)	0.964
spKt/V	1.34 (1.19–1.47)	1.35 (1.20–1.50)	0.557

Abbreviations: *AAC score* abdominal aortic calcification score, *DM* diabetes mellitus, *HTN* hypertension, *CAD* coronary artery disease, *BMI* body mass index, *ALT* alanine aminotransferase, *ALKP* alkaline phosphatase, *FBS* fasting blood sugar, *Calcium* serum total calcium (corrected by albumin), *iPTH* intact-parathyroid hormone, *spKt/V* Single-pool Kt/V

^a^Parametric variables (include serum cholesterol, calcium); except this two variables, all of other continuous variables else are nonparametric

**Table 4:** Related confounders of higher AAC score group (AAC score > 5.5) in multivariate linear regression

	Higher AAC score group	
	Beta	*p* value
Age(per 1 year old increase)	0.238	**< 0.001**
DM (Ref: no)	0.179	**0.006**
HTN (Ref: no)	0.002	0.975
CAD history (Ref: no)	0.177	**0.002**
Vintage (per 1 month increase)	0.189	**0.001**
BMI (per 1 kg/m^2^ increase)	−0.048	0.440
Creatinine(per 1 mg/dl increase)	−0.014	0.832
Fasting blood sugar (per 1 mg/dl increase)	0.009	0.885
Triglyceride(per 1 mg/dl increase)	−0.004	0.950
iPTH (per 1 pg/mL increase)	0.035	0.580
Calcium (per 1 mg/dl increase)	0.001	0.988
Phosphate (per 1 mg/dl increase)	0.084	0.170
spKt/V (per 1 unit increase)	−0.005	0.941

Abbreviations: *AAC score* abdominal aortic calcification score, *DM* diabetes mellitus, *HTN* hypertension, *CAD* Coronary artery disease, *iPTH* intact-parathyroid hormone, *spKt/V* Single-pool Kt/V

## Discussion

The major finding of our study is that higher AAC score can predict future CAD events in chronic HD patients. After adjustment of other known confounders, we found that the hazard ratios of AAC for CAD was 1.039 in multivariate Cox-regression model, *p* = 0.016. One point of AAC score increase was associated with 4% increase in the risk of future CAD events. The CAD-free survival rate was lower in high AAC score group (AAC score > 5.5) than in low AAC score group (AAC score < 5.5) with statistic significance (*p* = 0.001). We demonstrated that AAC score, independently of other contributive factors (prior history of CAD and higher BMI value), successfully predicted future CAD events in HD patients.

The most important contributive factors for future CAD occurrence in our study were prior history of CAD and BMI. In CKD population, patients with preexisting cardiovascular disease had been reported to be associated with an increased risk for recurrent cardiovascular outcome [12]. In our finding, patients with higher BMI were also associated with future CAD events. The majority (88%) of our study cases’ BMI ranged from normal to overweight by the BMI criteria for Asian populations [13]. The BMI distribution of our study subjects was as follows: 70% of patients’ BMI 18.5–25 (normal); 18% BMI 25–30 (overweight); 9% BMI < 18.5 (underweight); 3% BMI > 30 (obesity). Higher BMI had been reported to be associated with inflammation in HD patients [14], and was compatible with our finding that higher BMI, with a more severe inflammatory status, may increase the incidence of future CAD events.

In our cohort study, traditional cardiovascular risk factors, such as diabetes, baseline fasting blood sugar, serum phosphate level, did not influence the future coronary artery disease. Although the characteristics of these traditional parameters significantly increased the risk of CAD in univariate Cox regression model (Table 2, Model 1, crude hazard ratio), they did not statistically influence the risk of future cardiovascular disease after adjustment of all known confounders (Table 2, Model 2, adjusted hazard ratio). AAC score, however, significantly increased the multivariate hazard ratios in Cox-regression analysis.

In univariate model, hypocholesterolemia increased the risk for future CAD events, we though the major reason is related to the uremic inflammation status. Reverse epidemiology of cardiovascular events in maintenance HD patients had been reported [15]. In ESRD patients, hypocholesteremia reflected higher inflammation and likelihood of malnutrition inflammation. These patients tended to have higher CAD risk because of uremic inflammation. However, serum cholesterol appeared to not influence CAD prediction in our multivariate Cox regression analysis.

Recently, we have shown that a positive association between AAC score and new CAD event in ESRD patients on peritoneal dialysis [16]. In prior study, we found AAC score greater than 5.5 predicted future CAD events (*p* = 0.003, log-rank test in Kaplan Meier study). In this study, AAC score greater than 5.5 can also predict CAD in hemodialysis patients. Thus, we believe that the best cut-off value of AAC score is 5.5 to predict CAD in ESRD patients. In the previous study on peritoneal patients, the risk factors of higher AAC score were age, duration of dialysis, presence of diabetes, and history of cardiovascular disease [3, 17]. Our current study on hemodialysis patients, compatible with the results on peritoneal dialysis patients [16], also showed the above mentioned factors were associated with high AAC score.

Dyslipidemia with production of oxidatively modified LDL (or oxidized LDL, oxLDL) is the main hypothesis of CAD in general population. Macrophage uptakes oxLDL and triggers immune reaction. This inflammatory process leads to endothelial dysfunction, atherosclerosis, and clinical cardiovascular disease [18]. Therefore, oxidative stress with endothelial dysfunction has been known to be the major pathogenic factors associated with CAD [19, 20]. CAD is highly prevalent in ESRD patients and results in high mortality and poor long term survival [21]. CKD per se had long been proven to be associated with oxidative stress and endothelial dysfunction [22, 23]. The markedly increased oxidation and impaired endothelial function can also be seen in ESRD patients [24]. Treatment of dyslipidemia in hemodialysis patients, however, did not decrease the CAD incidence in these patient group (4D, AURORA, SHARP study) [25–27]. Factors other than dyslipidemia may be the CAD maker in hemodialysis patients.

Vascular calcification is also a manifestation of atherosclerosis and a cardiovascular risk factor in chronic kidney disease and uremia patients [28, 29]. The mechanism for atherosclerosis is quite different in CKD population. Mechanism of osteogenic transdifferentiation of vascular smooth muscle reported in CKD [30] contributed to medial layer vascular calcification [31]. As abdominal aortic calcification can be an indicator of vascular calcification, our results may implicate AAC as a marker of CAD in hemodialysis patients. By measuring the abdominal aortic calcification, we can sooner identify the higher risk CAD patients in patients on maintenance hemodialysis and may further prevent occurrence in these patients.

### Limitations

There are still some limitations in our study. First, this is a prospective observation cohort study in one single center. Second, we had a low prevalence of calcimimetic agent use (< 5%), an important issue in patients’ outcome with secondary hyperparathyroidism and mineral bone disease on maintenance hemodialysis. We cannot further analyze the effect on vascular calcification.

## Conclusions

AAC score can predict occurrence of new CAD events in chronic hemodialysis patients. The best cut-off value of AAC score is 5.5. AAC score greater than 5.5 is a reliable abdominal aortic calcification marker, and can predict future CAD in ESRD patients. In hemodialysis patients, major contributive factors for higher AAC score are advanced age, presence of diabetes, with prior history of CAD and longer dialysis vintage.

## Abbreviations

4D study: Die Deutsche Diabetes Dialyse Studie
AAC: Abdominal aortic calcification
ALKP: Alkaine phosphatase
ALT: Alanine aminotransferase
AURORA study: A Study to Evaluate the Use of Rosuvastatin in Subjects on Regular Hemodialysis: An Assessment of Survival and Cardiovascular Events
BMI: Body mass index
CAD: Coronary artery disease
CKD: Chronic kidney disease
CKD-MBD: Chronic kidney disease-mineral bone disease
DM: Dabetes mellitus
ESRD: End-Stage Renal Disease
FBS: Fasting blood sugar
HD: Hemodialysis
HTN: Hypertension
iPTH: Intact parathyroid hormone
KDIGO: The Kidney Disease Improving Global Outcome
LDL: Low-density lipoprotein
ROC: Receiver operation curve
SHARP study: Study of Heart and Renal Protection
spKt/V: Single-pool Kt/V
